# Nevoid follicular epidermolytic hyperkeratosis in a blaschkoid epidermal nevus

**DOI:** 10.1016/j.jdcr.2026.05.070

**Published:** 2026-06-17

**Authors:** Ahmed Sami Raihane, Le Wen Chiu, Hillary R. Elwood, John R. Durkin

**Affiliations:** a University of New Mexico School of Medicine, Albuquerque, New Mexico; b Department of Dermatology, University of New Mexico School of Medicine, Albuquerque, New Mexico; c TriCore Reference Laboratories, Albuquerque, New Mexico

**Keywords:** cutaneous mosaicism, epidermal nevus, epidermolytic hyperkeratosis, nevoid follicular epidermolytic hyperkeratosis, nevus unius lateralis, vulvar lesion

## Introduction

Epidermal nevi are congenital hamartomatous proliferations caused by postzygotic mutations that often follow the lines of Blaschko, reflecting cutaneous mosaicism.^1^ These lesions are usually present at birth and may evolve over time. Although most epidermal nevi are benign, diagnostic uncertainty may arise when lesions involve sensitive sites or show atypical clinical or histopathologic features.^2^ The most commonly identified postzygotic mutations in keratinocytic epidermal nevi involve RAS pathway genes, particularly HRAS and KRAS.^1^ Epidermolytic hyperkeratosis (EHK) is an uncommon histopathologic finding within epidermal nevi, estimated to occur in approximately 5% of cases,^3^ and reflects mosaic mutations in keratin 1 (KRT1) or keratin 10 (KRT10).^3^, ^4^, ^5^ Recognition of this pattern is clinically important due to potential implications for genetic counseling.^3^, ^4^, ^5^ Rarely, epidermolytic change may be localized predominantly or exclusively to the follicular epithelium, a pattern described as nevoid follicular epidermolytic hyperkeratosis.^6^ We present a case of follicular-confined EHK arising within a longstanding Blaschkoid epidermal nevus with progressive extension and vulvar involvement, highlighting this distinctive papular/follicular morphology and its unique histopathologic correlate.

## Case report

A 36-year-old woman with a congenital birthmark following the lines of Blaschko presented for evaluation of progressive lesion extension. The lesions were first evaluated in infancy, with a biopsy performed at 3 months of age. At that time, an evaluation for epidermal nevus syndrome was reportedly negative. The lesions remained stable throughout childhood and early adulthood and were treated with ammonium lactate.

Over time, the eruption slowly progressed but remained blaschkoid. Over the preceding 2 years, she noted extension involving the trunk, axilla, and extremities. Within the year preceding presentation, a new lesion developed on the left labia minora, prompting referral from gynecology for dermatologic evaluation and to rule out malignancy. The vulvar lesion was asymptomatic but appeared more hypopigmented and verrucous compared with her other lesions. The patient also expressed concern about fertility and the potential genetic implications of epidermolytic hyperkeratosis. Her family history was notable for melanoma in her father.

**Fig 1:**
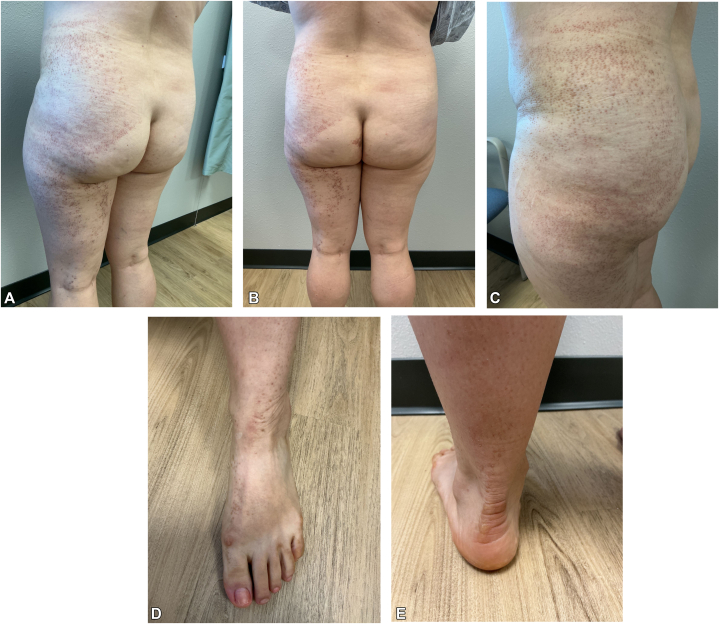
Clinical features of the patient. **A,** Posterior view of the trunk and lower extremities demonstrating unilateral blaschkoid plaques composed of monomorphic folliculocentric papules over the right flank, buttock, and thigh. **B,** Closer posterior view displays the sharply demarcated blaschkoid distribution over the right gluteal region and posterior thigh. **C,** Close-up view of the right flank demonstrating densely packed folliculocentric papules with a rough, keratotic surface. **D,** Distal lower extremity involvement with folliculocentric papules extending to the ankle and dorsal foot. **E,** Posterior ankle and heel showing continued folliculocentric papules without ulceration or fissuring.

Cutaneous examination revealed an extensive blaschkoid distribution of monomorphic, rusty to skin-colored follicular scaly papules extending from the right flank and hip over the buttock, posterior thigh, and calf to the ankle and dorsal foot (Fig 1, *A*-*E*). Additional involvement was present in the left axilla and medial upper extremity. The lesions appeared centered on hair follicles with relative sparing of intervening skin, giving a keratosis pilaris–like appearance rather than the confluent plaques seen in epidermal nevi. Given this atypical morphology, a biopsy was obtained.

**Fig 2:**
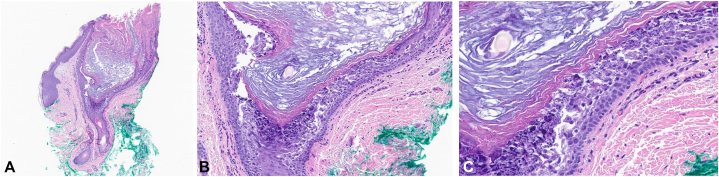
Histopathological features. **A,** Punch biopsy from the left axilla demonstrating epidermal acanthosis with hyperkeratosis and an associated dilated hair follicle. **B,** Higher-power view showing epidermolytic hyperkeratosis with perinuclear vacuolization and coarse keratohyalin granules involving the follicular epithelium. **C,** High-power view highlighting epidermolytic change within the follicular epithelium with adjacent follicular keratinization. (H**em**atoxylin-eosin stain; original magnifications: **A,** 40×; **B,** 100×; **C,** 200×).

Given the patient’s concern about progression and desire to avoid vulvar biopsy, a punch biopsy was obtained from a lesion on the left axilla. Histopathology demonstrated epidermal acanthosis with compact hyperkeratosis and prominent epidermolytic hyperkeratosis (EHK) confined to the follicular epithelium, without involvement of the interfollicular epidermis (Fig 2, *A*-*C*), consistent with a follicular epidermal nevus with EHK. Importantly, the biopsy demonstrated epidermolytic alteration with coarse keratohyalin granules and vacuolar degeneration of keratinocytes without suprabasal acantholysis or the dyskeratotic corps ronds and grains characteristic of Darier disease, supporting EHK over focal acantholytic dyskeratosis as the underlying pattern. A subsequent vulvar biopsy performed by gynecology showed similar findings without dysplasia or malignancy, supporting involvement by the same process. The patient was counseled on the benign nature of the lesions and the mosaic implications of EHK, and management was continued with topical keratolytics.

## Discussion

This case presents a rare and distinctive variant of epidermal nevus characterized by a papular, folliculocentric clinical morphology resembling keratosis pilaris along the lines of Blaschko, with the unique histopathologic correlate of EHK confined exclusively to the follicular epithelium, a pattern termed nevoid follicular epidermolytic hyperkeratosis. Progressive blaschkoid extension and vulvar involvement also posed diagnostic challenges and warranted histopathologic evaluation to exclude malignancy.

Epidermolytic hyperkeratosis (EHK) within an epidermal nevus is an uncommon histopathologic finding and represents a form of cutaneous mosaicism most often associated with postzygotic mutations in KRT1 or KRT10.^3^, ^4^, ^5^^,^^7^ KRT1-associated mutations have been described in epidermal nevi with EHK and are more frequently associated with palmoplantar keratoderma, whereas KRT10-associated mutations typically lack palmoplantar involvement.^3^, ^4^, ^5^^,^^7^ Recognition of mosaic EHK is clinically important because gonadal mosaicism and transmission to offspring have been reported, warranting genetic counseling.^3^, ^4^, ^5^^,^^7^

A distinctive feature of this case was the exclusive involvement of the follicular epithelium with sparing of the interfollicular epidermis. Clinically, this correlated with monomorphic follicular papules resembling keratosis pilaris distributed along the lines of Blaschko rather than the more typical confluent plaques of epidermal nevi. To our knowledge, epidermal nevus with EHK confined to the follicular epithelium has been reported only once previously, in the 1975 report by Plewig and Christophers describing “nevoid follicular epidermolytic hyperkeratosis.”^6^ This case represents a rare and likely underrecognized variant of epidermal nevus with EHK and suggests a mosaic keratin mutation preferentially affecting follicular keratinocytes.

Although malignant transformation within epidermal nevi is rare, squamous cell carcinoma and basal cell carcinoma arising within epidermal nevi have been reported.^8^^,^^9^ In this patient, histopathologic evaluation of both cutaneous and vulvar lesions demonstrated no evidence of dysplasia or malignancy. Of note, mosaic Darier disease was considered in the differential diagnosis given the blaschkoid papular morphology; however, the histopathologic findings were more consistent with EHK than focal acantholytic dyskeratosis. Specifically, the biopsy demonstrated epidermolytic alteration with coarse keratohyalin granules and vacuolar degeneration without the suprabasal acantholysis and dyskeratosis characteristic of Darier disease. This case underscores the diagnostic importance of clinicopathologic correlation in atypical blaschkoid eruptions. The finding of mosaic EHK carries reproductive implications: gonadal mosaicism may allow transmission of a pathogenic keratin mutation to offspring as generalized epidermolytic hyperkeratosis, making genetic counseling essential for affected individuals of reproductive age.^3^, ^4^, ^5^^,^^7^ The absence of genetic confirmation represents a limitation of this report; KRT1 or KRT10 sequencing of lesional tissue would provide definitive molecular support for the diagnosis.

## Conflicts of interest

None disclosed.
